# Combinations of ATR, Chk1 and Wee1 Inhibitors with Olaparib Are Active in Olaparib Resistant *Brca1* Proficient and Deficient Murine Ovarian Cells

**DOI:** 10.3390/cancers14071807

**Published:** 2022-04-01

**Authors:** Michela Chiappa, Federica Guffanti, Martina Anselmi, Monica Lupi, Nicolò Panini, Lisa Wiesmüller, Giovanna Damia

**Affiliations:** 1 Department of Oncology, Istituto di Ricerche Farmacologiche Mario Negri IRCCS, 20156 Milan, Italy; michela.chiappa@marionegri.it (M.C.); federica.guffanti@marionegri.it (F.G.); martina.anselmi@unimi.it (M.A.); monica.lupi@humanitasresearch.it (M.L.); nicolo.panini@marionegri.it (N.P.); 2 Department of Obstetrics and Gynecology, Ulm University, 89075 Ulm, Germany; lisa.wiesmueller@uni-ulm.de

**Keywords:** ovarian carcinoma, homologous recombination, PARPi, olaparib resistance

## Abstract

**Simple Summary:**

Poly(ADP-ribose) polymerases inhibitors (PARPis), including olaparib, have been recently approved for ovarian carcinoma treatment and PARPi resistance has already been observed in the clinics. With the aim of dissecting the molecular mechanisms of PARPi resistance, we generated olaparib resistant cells lines, both in a homologous recombination (HR)-deficient and -proficient background by continuous in vitro drug treatment. In the HR proficient background, olaparib resistance was caused by overexpression of multidrug resistance 1 gene (*MDR1*), while multiple heterogeneous co-existing mechanisms were found in olaparib resistant HR-deficient cells, including overexpression of *MDR1*, a decrease in PARP1 protein level and partial reactivation of HR repair. We found that combinations of ATR, Chk1 and Wee1 inhibitors with olaparib were synergistic in sensitive and resistant sublines, regardless of the HR status. These new olaparib resistant models will be instrumental to screen new therapeutic options for PARPi-resistant ovarian tumors.

**Abstract:**

Background. Poly(ADP-ribose) polymerases inhibitor (PARPi) have shown clinical efficacy in ovarian carcinoma, especially in those harboring defects in homologous recombination (HR) repair, including *BRCA1* and *BRCA2* mutated tumors. There is increasing evidence however that PARPi resistance is common and develops through multiple mechanisms. Methods. ID8 F3 (HR proficient) and ID8 *Brca1-/-* (HR deficient) murine ovarian cells resistant to olaparib, a PARPi, were generated through stepwise drug concentrations in vitro. Both sensitive and resistant cells lines were pharmacologically characterized and the molecular mechanisms underlying olaparib resistance. Results. In ID8, cells with a HR proficient background, olaparib resistance was mainly caused by overexpression of multidrug resistance 1 gene (*MDR1*), while multiple heterogeneous co-existing mechanisms were found in ID8 *Brca1-/-* HR-deficient cells resistant to olaparib, including overexpression of MDR1, a decrease in PARP1 protein level and partial reactivation of HR repair. Importantly, combinations of ATR, Chk1 and Wee1 inhibitors with olaparib were synergistic in sensitive and resistant sublines, regardless of the HR cell status. Conclusion. Olaparib-resistant cell lines were generated and displayed multiple mechanisms of resistance, which will be instrumental in selecting new possible therapeutic options for PARPi-resistant ovarian tumors.

## 1. Introduction

Ovarian cancer (OC) is one of the leading causes of gynecological cancer-related death [1]. The high-grade serous carcinoma (HGSOC) is the most common histological subtype [2]. Due to the relatively asymptomatic nature of OC and the lack of adequate screening tests, approximately 75% of patients are diagnosed with advanced disease, resulting in a poor five-year overall survival [3]. The standard treatment of OC consists of optimal cyto-reductive surgery followed by adjuvant platinum/taxol-based chemotherapy, which have improved the overall survival with 70% of patients achieving complete remission after first-line therapy [4]. Unfortunately, about half of the patients will relapse with a resistant tumor [5].

In the last decade, a better characterization of the molecular and biological features of HGSOC have paved the way to new therapeutic approaches, including poly(ADP-ribose) polymerase inhibitors (PARPi) [6]. According to the Cancer Genome Atlas (TCGA) consortium, more than half of HGSOC have homologous recombination (HR) deficiency due to mutations in genes involved in this pathway, including *BRCA1* and *2* [7]. The synthetic lethality between HR deficiency and PARP inhibition [8,9] is at the basis of the strong antitumor activity of PARPi in HR-deficient tumors [10,11]. PARP enzymes catalyze the addition of poly(ADP-ribose) adducts to target proteins involved in cell signaling and DNA damage response, and their inhibition leads to accumulation of DNA single strand breaks (SSBs) and to a stall of replication forks, eventually progressing to double strand breaks (DSBs), which are highly cytotoxic to cells lacking a proficient HR [12]. PARPi also trap PARP1 into DNA, forming PARP-DNA complexes, causing replication stress and replication fork collapse [13]. Notably, has been shown that these PARP-DNA complexes correlate with the cytotoxic activity of PARPi and need PARP1 protein [14].

These data have been recently challenged by the hypothesis that the determinants of PARPi toxicity may be the single strand DNA gaps resulting from the loss of PARP1 and BRCA1/2 [15,16]. Regardless the precise mechanism of action, however these findings modified the therapeutic armamentarium for *BRCA1/2* deficient ovarian carcinoma over recent years [6]. In 2014, the FDA approved the use of olaparib (AZD-2281) as maintenance therapy for platinum-sensitive *BRCA*-mutated HGSOC [17] and after that two other PARPi, rucaparib [18] and niraparib [19], were approved. Although PARPi were first indicated for relapsing, platinum-sensitive *BRCA* mutated cancers, some clinical advantages have been reported in HR-proficient HGSOC and these drugs are now approved for maintenance therapy after front line platinum-based chemotherapy [20,21,22].

Despite their clinical benefits, PARPi treatment is still associated with the development of resistance [23]. Understanding the mechanisms of intrinsic and acquired PARPi resistance (mainly in a HR-deficient background) is a clinical need and of great importance to improve their efficacy in particular in the HR-proficient HGSOC where effective target therapies are lacking, and to possibly delay the development of resistance. To the best of our knowledge, no syngeneic murine olaparib resistant models are available in a HR- proficient and -deficient background. These models would be, in our opinion, extremely important tools to better define and compare the mechanisms driving olaparib resistance. For this reason, we generated and characterized olaparib-resistant murine HGSOC cell lines (ID8) in a HR-deficient and -proficient background. The main mechanism of PARPi resistance in the HR-proficient background was the overexpression of MDR1, while various co-existing mechanisms were found in HR deficient cells resistant to olaparib (overexpression of MDR1, decrease in PARP1 protein level, and partial restoring of HR function). In addition, different combinations of DNA Damage Response (DDR) agents (i.e., ATR, Chk1 and Wee1 inhibitors) with olaparib were synergistically active in both sensitive and resistant sublines, regardless their HR status.

## 2. Materials and Methods

### 2.1. Olaparib-Resistant Clones

ID8 F3 (*Trp53-/-*) and ID8 *Brca1-/-* (*Tp53-/-; Brca1-/-*), indicated here, respectively, as F3 and *Brca1-/-*, were kindly provided by I.A. McNeish (Institute of Cancer Sciences, University of Glasgow, Glasgow, UK) [24] and maintained in DMEM medium (Gibco, Life Technologies. Carlsbad, CA, USA) supplemented with 10% glutamine, 4% FBS, 5 µg/mL insulin, 5 µg/mL transferrin and 5 ng/mL sodium selenite, at 37 °C with 5% CO_2_. Olaparib resistance was induced by treating cells continuously with increasing doses of the drug (from 10 µM to 30 µM for ID8 F3 and from 0.2 µM to 2 µM for ID8 *Brca1-/-*). As illustrated in Figure S1, olaparib sensitivity was tested once a month and after six months, ID8 F3 olaparib-resistant (F3 OlaR) and the ID8 *Brca1-/-* olaparib-resistant (*Brca1-/-* OlaR) cell lines were obtained.

### 2.2. Cell Growth

Growth curves were obtained by seeding the cells at 1000 cells/mL in 96-well plates and proliferation was examined with the MTS assay (Promega, Madison, WI, USA) at different time points. For the colony assay, cells were seeded at 150 cells/mL in six-well plates; colonies were left to grow for ten days, and then stained with Gram’s Crystal Violet solution (Merck, Darmstadt, Germany). The number of colonies were quantified using the QICAM 32–0030C camera (QIMAGING, Surrey, BC, Canada) and Colony plus 2.0 program.

### 2.3. Flow Cytometry

For the DNA content analyses, exponentially growing cells treated or not with olaparib at the time points indicated were washed twice in ice-cold PBS and fixed in ice-cold 70% ethanol, washed again in PBS, re-suspended in 2 mL of a solution containing 25 μg/mL of propidium iodide (PI) and 25 μL of RNase 1 mg/mL, and stained overnight at 4°C in the dark. For the apoptosis assay, 500.000 cells were stained with the FITC Annexin V/PI Apoptosis Detection Kit (BioLegend, San Diego, CA, USA). FACS Calibur (Becton Dickinson, Franklin Lakes, NJ, USA) was used for analysis, as reported [25].

### 2.4. Quantitative Reverse Transcription-(RT)-PCR

Total RNA from cells was purified with the Maxwell 16 LEV SimplyRNA (Promega, Madison, WI, USA) and retro-transcribed with the High-Capacity cDNA Reverse Transcription kit (Applied Biosystems, Waltham, MA, USA). Gene expression was measured by quantitative real-time PCR with SYBR green technology (Applied Biosystems) using ad hoc-designed primers (Table S1). The real time-PCRs were run in triplicate. All data were normalized to the levels of the *β-actin* gene and analyzed using the ΔΔCt method.

### 2.5. Drugs and Treatments

Olaparib was purchased from TargetMol; rucaparib, niraparib, KU55933 (ATM inhibitor), AZD6738 (ATR inhibitor), AZD7762 (Chk1 inhibitor) and AZD1775 (Wee1 inhibitor) from Axon Medchem; cisplatin from Sigma Adrich; carboplatin from Adipogen; paclitaxel from ChemieTek; doxorubicin and verapamil from Merck. All the drugs were dissolved in DMSO as stock solutions and diluted in medium just before treatment. For cytotoxicity experiments, cells were seeded at 1000 cells/mL and treated with different drug concentrations in 96-well plates 48 h after seeding. After five days, cell viability was examined with the MTS assay system (Promega) and absorbance was acquired using a plate reader (GloMax Discover, Promega). Drug concentrations inhibiting growth in 50% of the cells (IC_50_) were calculated for each cell line, with the interpolation method on Prism 8.3.0 (GraphPad Software).

### 2.6. cDNA Sequencing

Total RNA from *Brca1-/-* and *Brca1-/-* OlaR cells was purified and retro-transcribed as described before. The cDNA was amplified with PCR using seven pairs of specific primers to bind the whole *PARP1* sequence (Table S1). Amplified PARP1 bands were separated by electrophoresis, and Sanger-sequenced.

### 2.7. Western Blot

Cell pellets were lysed for 30 min in ice-cold whole cell extract buffer (50 mM TrisHCl pH 7.4, 250 mM NaCl, 0.1% Nonidet NP40, 5 mM EDTA, 50 mM NaF and a protease inhibitor cocktail (Sigma-Aldrich, St. Louis, MO, USA). Lysates were cleared by centrifuging at 12,000 rpm for 5 min and the protein concentration was determined using a BioRad assay kit (BioRad, Hercules, CA, USA). Cell lysates (50µg) were resolved on 10–12% SDS-PAGE (polyacrylamide gel electrophoresis) gels. Proteins were then transferred to nitrocellulose membranes (Merck Millipore, Burlington, MA, USA). Immunoblotting was carried out with the following antibodies: rabbit anti-PARP #9542 (cell signaling, 1:1000), rabbit anti-γH2AX #9718 (Cell Signaling, Danver, MA, USA, 1:1000), goat anti-ACTIN sc-1615 (Santa Cruz Biotechnology, Dallas, TX, USA, 1:500) and mouse anti-RAN sc-271376 (Santa Cruz Biotechnology, 1:500). The secondary antibodies conjugated with horseradish peroxidase (HRP) anti-rabbit #1706515 and anti-mouse #1706516 were purchased from BIO-RAD Laboratories S.r.l., anti-goat sc-2354 from Santa Cruz Biotechnology. HRP substrate (ECL Western Blotting Detection, Amersham-Life Science, Amersham, UK) was added and the signal was detected with the Odyssey Fc instrument (Li-COR). All the uncut filter and relative densitometric raw data have been included in Figure S7.

### 2.8. RAD51 Immunofluorescence

Cells were seeded on coverslips in 24-well plates at 15,000 cells/mL and treated after 24 h with olaparib (IC_50_ dose for 24 h) or irradiated (10 Gy), then fixed in 5% paraformaldehyde for 30 min. Cells were permeabilized with 0.2% Triton (Sigma) in PBS for 15 min and stained with rabbit anti-RAD51 ab63801 (Abcam, Cambridge, UK) diluted 1:1000 in blocking solution (BSA 5%). Nuclei were stained with 4′,6-diamidino-2-phenylindole (DAPI) (30 ng/mL in PBS, Sigma-Aldrich, St. Louis, MO, USA). Slides were mounted with Vectashield solution (VectorLab) and observed using a Nikon Instruments A1 Confocal Laser Microscope, with the Plan Fluor 40 × DIC M N2 NA = 0.8 WD = 660 uM objective. RAD51 foci were quantified by scoring cells with five or more foci per nucleus. Five areas in z-stacking of each sample were acquired and analyzed with ImageJ FIJI win-64 software.

### 2.9. DNA Repair

Pathway-specific DSB repair activities were investigated by using functional EGFP-based assays [26]. Briefly, *Brca1-/-* and *Brca1-/-* OlaR cells were co-transfected with different plasmid mixtures containing the meganuclease expression plasmid (pCMV-I-Sce1) together with one of the recombination substrates (HR-EGFP/3′EGFP and EJ5 SceGFP) [27,28] and the wtEGFP plasmid in split samples for the determination of transfection efficiency. Cells were nucleofected using the Cell Line Nucleofector Kit V (Amaxa/Lonza), then seeded in six-well plates. After 24 h cellular fluorescence was quantified by flow cytometry (FACS Calibur, Becton Dickinson, San Jose, California, USA) and recombination frequencies were calculated from the fraction of wtEGFP positive cells normalized for transfection efficiency (20–50%).

### 2.10. Statistical Analysis

Statistical analysis was carried out with GraphPad Prism 8.3.0 (GraphPad Software), using the tests specified in the legends to the figures.

## 3. Results

### 3.1. In Vitro Generation of Olaparib-Resistant Sublines

**Figure 1:**
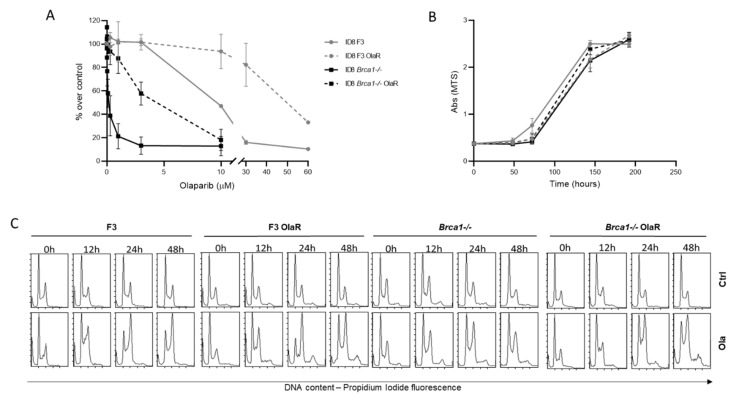
Characterization of F3, F3 OlaR, *Brca1-/-* and *Brca1-/-* OlaR cells. (**A**) Olaparib-dose response curve in F3 (grey line), F3 OlaR (dashed grey line), *Brca1-/-* (black line) and *Brca1 -/-* OlaR cells (dashed black line). Data are the mean ± standard deviation (SD) of four independent experiments. (**B**) Growth curves in F3 (grey line), F3 OlaR (dashed grey line), B *Brca1*-/- (black line) and B *Brca1-/-* OlaR cells (dashed black line) at different times from seeding. Data are the mean ± SD of six replicates. (**C**). Flow cytometric analysis of DNA content in cells in F3, F3 OlaR, *Brca1-/-* and *Brca1-/-* OlaR cells treated or not with olaparib at different times (12 h, 24 h and 48 h) with the corresponding drug IC_50_.

We generated olaparib-resistant ovarian carcinoma cells in a HR-deficient and a HR-proficient background. We used murine ID8 F3 (*Trp53−/−*) [29] and ID8 *Brca1-/-* (*Trp53-/-; Brca1-/-*) [24] cells; the ID8 *Brca1-/-* are 75 times more sensitive to olaparib than the F3 (Figure 1A), as already reported [24]. As detailed in Material and Methods, after exposing cells to increasing concentrations of continuous olaparib, we established the F3 OlaR subline from ID8 F3, seven times more resistant (IC_50_ 88.59 ± 22.11 vs 12.99 ± 2.31 µM, *p* = 0.0016) and *Brca1-/-* OlaR subline from ID8 *Brca1-/-*, 24 times more resistant (IC_50_ 3.94 ± 0.55 vs 0.17 ± 0.028 µM, *p* < 0.0001) than parental cells (Table 1 and Figure 1A). The dose response curve in sensitive and resistant cells by colony assay confirmed olaparib resistance (Figure S2). There were no differences in cell growth between the parental and the resistant sublines. (Figure 1B). For both resistant lines, resistance was maintained for six months, culturing the cells in drug-free medium. In addition, in the same period, no changes in cell growth and morphology were detected in resistant cells compared to parental cells, in both *Brca1* proficient and deficient backgrounds.

### 3.2. Cell Cycle Effects and Olaparib Sensitivity

To better characterize the resistant cell lines, cell cycle patterns after treatment with olaparib were studied by flow cytometric analysis of DNA content (Figure 1C). Cells treated or not with the corresponding olaparib IC_50_ were analyzed at different time points (12, 24 and 48 h) from the beginning of treatment. In both F3 and *Brca1-/-* cells, olaparib caused a G2/M block that persisted for up to 48 h. In *Brca1-/-* cells a polyploid subpopulation appeared at 12 h of treatment and increased over time. This subpopulation masks the true cell cycle perturbation, as G2/M cells of the diploid population may overlap with the G1 phase of the polyploid cells. Nevertheless, HR-deficient cells seem to lack an efficient block in G2/M, allowing them to somehow progress through the cell cycle without properly undergoing optimal cell division. In both resistant cell lines, there was a lower G2/M block at 12 h compared to parental cells, and this seemed to increase at 24 and 48 h. There was no difference in apoptosis induction in F3, *Brca1-/-* and their corresponding resistant sublines (Table S2).

### 3.3. Pharmacological Characterization

To investigate the mechanisms of olaparib resistance, we pharmacologically characterized our resistant cell lines and compared them to the parental ones. Only *Brca1-/-* OlaR were cross-resistant to both rucaparib (IC_50_ of 1.03 ± 0.11 vs 0.064 ± 0.015 µM, respectively,) and niraparib (IC_50_ 0.284 ± 0.035 vs 0.065 ± 0.012 µM), while the F3 OlaR cells were equally responsive to niraparib (IC_50_ 1.00 ± 0.19 vs 1.11 ± 0.22 µM) and more sensitive to rucaparib (IC_50_ 4.05 ± 0.61 vs 12.5 ± 2.95 µM) than the parental cells (Table 1 and Figure S3). Platinum compounds (cisplatin and carboplatin) were similarly active in sensitive and resistant lines; doxorubicin displayed cross-resistance in both Ola-resistant sublines, while paclitaxel was cross-resistant only in F3 OlaR. Yondelis (ET-743), a marine-derived tetrahydroisoquinoline alkaloid with antitumor activity [30], was cross-resistant in *Brca1-/-* OlaR and had similar activity in F3 and F3 OlaR cells (Table 1 and Figure S3).

Considering the stronger dependence on DNA damage response (DDR) pathways [31,32] reported in PARPi resistant cells, we tested the cytotoxic activities of some DDR inhibitors, i.e., inhibitors of ATR, Chk1 and Wee1 kinases involved in the DDR pathways [33]. All these inhibitors were much more active in *Brca1-/-* cells than in F3 cells, confirming the greater dependence of cells with a defective HR on these DDR pathways [34]. Both resistant sublines were less sensitive (from 2 to 4 times) to ATR and Chk1 inhibitors (AZD6738 and AZD7762); the Wee1 inhibitor (AZD1775) showed cross resistance in *Brca1-/-* OlaR, but not in the F3 OlaR cells, while cytotoxic activity was similar with the ATM inhibitor KU55933 (Table 1, Figure S3).

### 3.4. MDR and PARP1 Levels as Determinants of Olaparib Resistance

**Figure 2:**
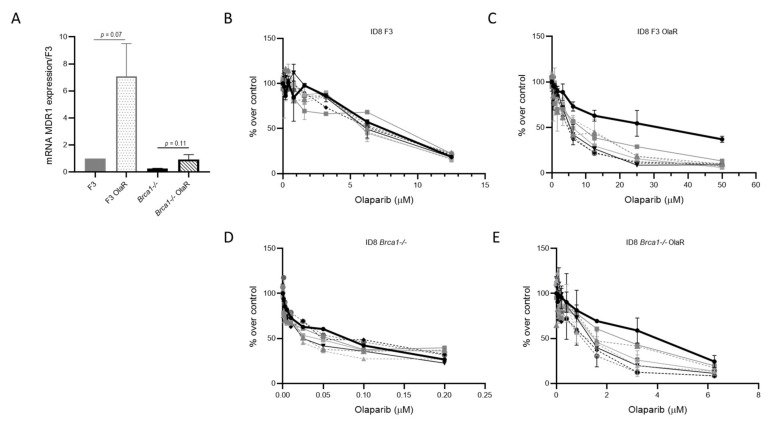
*MDR1* gene expression and verapamil treatment. (**A**) MDR1 mRNA levels measured by RT-PCR. Actin normalized MDR1 expression in F3 (filled grey column), F3 OlaR (patterned grey column), *Brca1-/-* (filled black column) and *Brca1-/-* OlaR cells (patterned black column). Values correspond to the mean of three independent experiments, carried out in triplicate and are expressed as fold-change over the F3 cells. For statistical analyses unpaired *t*-test was used. (**B**–**E**) Olaparib-dose response curve of F3 (panel **B**), F3 OlaR (panel **C**), *Brca1-/-* (panel **D**) and *Brca1-/-* OlaR cells (panel **E**) alone (bold black line) or in combination with verapamil 0.375 nM (dark grey line), 0.75 nM (dashed dark grey line), 1.5 nM (light grey line), 3 nM (dashed light grey line), 6 nM (black line), 12 nM (dashed black line). Data are the mean ± SD of three independent experiments.

MDR1 has been associated with PARPi resistance in both the HR-proficient and -deficient backgrounds [35,36]. The patterns of cross-resistance with paclitaxel and doxorubicin (Table 1) suggested the possible involvement of the MDR1 pump efflux in olaparib resistance, as both drugs are MDR1 substrates and its overexpression has been associated with drug resistance. We measured the MDR1 mRNA expression levels in sensitive and resistant cells by RT-PCR (Figure 2A). Both OlaR sublines showed upregulation of mRNA levels compared to the parental ones, respectively, 7- and 3.7- fold in F3 OlaR and *Brca1-/-* OlaR.

To clarify the exact role of MDR1, we explored the effect of verapamil (a calcium channel blocker that inhibits the transport function of MDR1 [37]) on olaparib cytotoxicity. Co-treatment with verapamil (at doses higher than 3 nM, not causing cell cytotoxicity; Figure 2) re-sensitized the F3 OlaR subline to the level of the parental cells (Figure 2B,C, IC_50_ 24.62–5.82 µM), while olaparib resistance was only partially restored in Brca1-/- OlaR cells (Figure 2D and E IC_50_ 3.37–1.25 µM). The combination did not increase olaparib cytotoxicity in the parental cells (Figure 2B,D).

**Figure 3:**
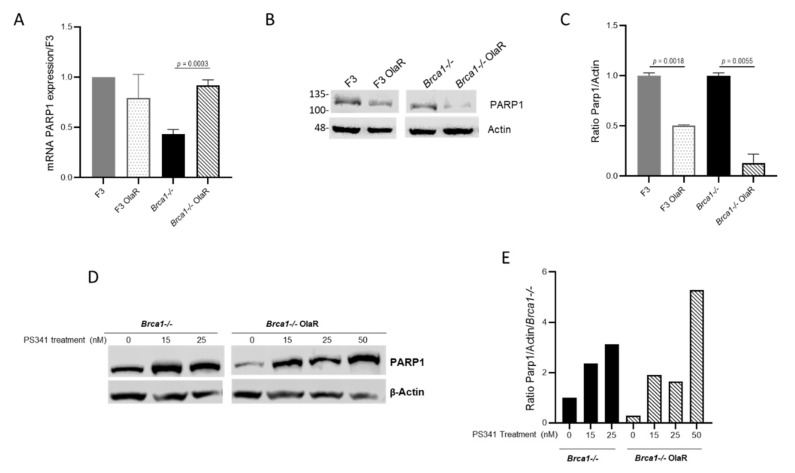
PARP1 expression in F3, F3 OlaR, *Brca1-/-* and *Brca1-/-* OlaR cells. (**A**) PARP1 mRNA expression measured by RT-PCR and normalized by actin mRNA in F3 (filled grey column), F3 OlaR (patterned grey column), *Brca1-/-* (filled black column) and *Brca1-/-* OlaR cells (patterned black column). Values are the mean ± SD of three independent experiments, carried out in triplicate and are expressed as fold-change over the corresponding sensitive cells. (**B**) PARP1 protein in F3, F3 OlaR, *Brca1-/-* and *Brca1-/-* OlaR cells detected by western blot analysis. Actin was included as internal loading control. (**C**) Densitometric analysis of the western blot results expressed as the ratio of PARP1 to actin level. (**D**) PARP1 western blot analysis in cells treated or not with different doses of the proteasome inhibitor PS341 for 8 h. (**E**). Densitometric analysis of the PARP1 western blot results. Data are expressed as the ratio of PARP1 to actin level over the *Brca1-/-* PARP1 to actin ratio. For statistical analyses unpaired *t*-test was used.

As PARP1 is the main target of PARPi and its lack has been associated with olaparib resistance in a HR deficient background [38], we examined its expression. In F3 and F3 OlaR PARP1 mRNA was equally expressed, while a decrease in its protein level could be observed in F3 OlaR; Brca1-/- OlaR showed doubling of the mRNA (Figure 3A), but clear downregulation of the protein (Figure 3B,C). Sequencing the *PARP1* gene revealed no mutation that could justify the increased instability [38] (data not shown). We reasoned that the PARP1 protein in ID8 *Brca1-/-* OlaR might be more instable and/or more easily degraded. We treated cells with PS341, a proteasome inhibitor, and the protein became stable in both sensitive and resistant ID8 *Brca1 -/-* cells (Figure 3D,E), suggestive of a faster protein turnover in the latter. Slight increase in PARP1 protein level was observed after 48hrs olaparib treatment, in both the resistant sublines (Figure S3).

### 3.5. DNA Repair

**Figure 4:**
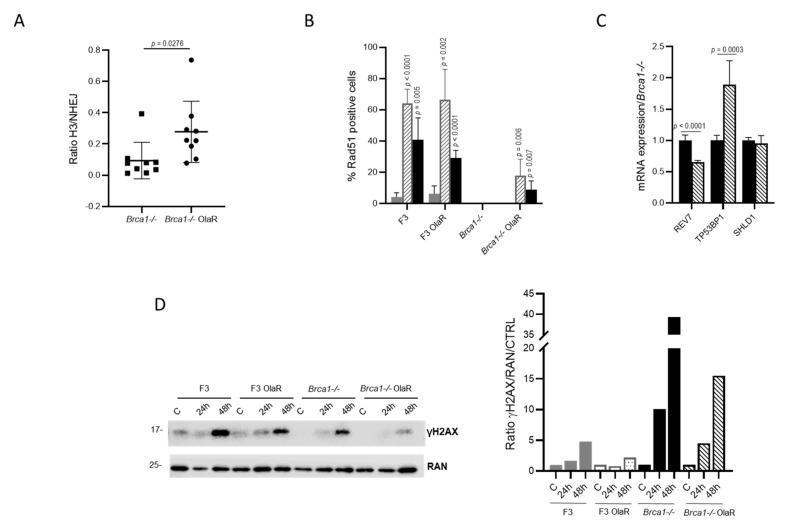
Homologous recombination repair in olaparib-sensitive and -resistant cells. (**A**). Basal levels of homologous repair and NHEJ by analyzing pathway-specific DSB repair of GFP-based reporter plasmids HR-EGFP/3′EGFP and EJ5SceGFP transfected in the sensitive and resistant cells with the I-*Sce*I meganuclease expression plasmid as detailed in Material and Methods. Data are expressed as the ratio between homologous repair (HR plus single-strand annealing, SSA) and NHEJ efficiency; each dot represents a single experimental point. (**B**). Percentage of RAD51 positive cells treated or not with olaparib (filled black column) and IR (patterned grey column) at 24 h. A representative experiment is shown. For statistical analyses of treated cells over the control unpaired *t*-test was used (**C**). mRNA expression of REV7, TP53BP1 and SHLD1 in *Brca1-/-* (filled black column) and *Brca1-/-* OlaR (patterned black column). Values are normalized by actin mRNA expression and are the mean of three independent experiments, run in triplicate, and are expressed as fold-change over *Brca1-/-* values. (**D**). Western blot analysis of γH2AX after olaparib treatment and its densitometric quantification. For statistical analyses unpaired *t*-test was used.

The restoration of the HR is one of the mechanisms of resistance to PARPi in a HR deficient background [39]. We therefore examined the basal levels of homologous repair as compared to NHEJ by analyzing pathway-specific DSB repair of GFP-based reporter plasmids HR-EGFP/3′EGFP and EJ5SceGFP transfected in the sensitive and resistant cells along with the I-*Sce*I meganuclease plasmid, as detailed in Material and Methods. These repair substrates monitor homologous repair and NHEJ following I-*Sce*I-mediated cleavage [40]. There was an increase-even if not statistically significant- in the percentage of homologous repair efficiency in *Brca1-/-* OlaR compared to *Brca1-/-* cells (Figure S5) and a significant decrease in NHEJ repair, which translated into a statistically significant higher homologous repair/NHEJ ratio (Figure 4, panel A).

Generally, the lack of HR is associated with an increase in NHEJ, as our data would suggest [41]. The fact that the homologous repair/NHEJ ratio in resistant cells rose indirectly suggests that either HR and/or the compensatory pathway single-strand annealing (SSA) rose, in agreement with the increase in RAD51 positivity detectable in *Brca1-/-* OlaR cells.

RAD51 foci induction in response to DNA damage is a recognized functional test for HR proficiency [42]. While in ID8 *Brca1-/-* no RAD51 foci could be seen at baseline (control cells) nor after damage, their number increased after IR and olaparib treatment in the corresponding resistant cells. Yet, the damage induced RAD51 levels were still lower than in F3 and F3 OlaR cells, where no difference in RAD51 foci formation was detected at either baseline or after IR and olaparib treatments (Figure 4B). The partial restoration of HR was not due to a lower mRNA levels in 53BP1 and SHLD1, even though there was a significant reduction in REV7 mRNA (Figure 4C). When we tested the induction of γH2AX after olaparib treatment, both sensitive and resistant cells showed a clear induction. A lower γH2AX induction could be observed in olaparib resistant cells than in the corresponding sensitive cells (Figure 4D). In aggregate, these data suggest that in ID8 *Brca1-/-* OlaR HR was partially restored.

### 3.6. DDR Inhibitors and Olaparib Combinations

**Figure 5:**
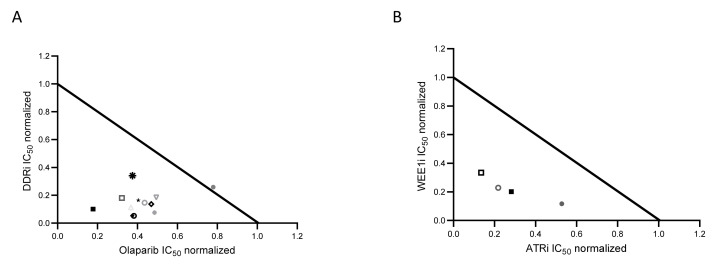
Synergistic combinations of DDR agents and olaparib. (**A**) Normalized IC_50_ isobolograms showing the synergistic effects of the combination of olaparib with the inhibitors of ATR (F3🞇; F3 OlaR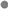; *Brca1-/-* 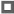; *Brca1-/-* OlaR 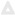), Chk1 (F3 ◆; F3 OlaR 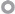; *Brca1-/-* 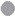; *Brca1-/-* OlaR ★) and Wee1 (F3 🞿; F3 OlaR ■; *Brca1-/-* 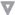; *Brca1-/-* OlaR 🞛). (**B**) Normalized IC_50_ isobolograms showing the synergistic effects of the combination of ATRi AZD6738 and the WEE1i AZD1775 in F3 (●), F3 OlaR (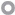), *Brca1-/-* (■) and *Brca1-/-* OlaR (🞑). Data are the mean ± SD of three independent experiments.

DDR inhibitors are reported to be very active in combination treatments, in vitro and in vivo ovarian cancer models [43], and in some cases can overcome PARPi resistance (for a recent review see [44,45]). Indeed, early clinical trials of PARPi combined with Wee1, Chk1 and ATR inhibitors have been explored in different subsets of OC patients [46,47,48,49,50]. We tested the combination of Wee1, Chk1 and ATR inhibitors with olaparib in our models (Figure 5 and Figure S6). The combinations were synergistic in both sensitive parental and olaparib resistant cell lines, except for the combination of ATRi and olaparib in the F3 OlaR cells, that seemed additive. In addition, the combination of ATRi and Wee1 was synergistic in both sensitive and resistant HR-proficient and deficient ID8 cells (Figure 5B).

## 4. Discussion

With the approval of PARPi in both front-line and recurrent settings, including HGSOC [24,51], PARPi resistance is an emerging clinical problem. While HR deficiency is generally a biomarker of sensitivity, HR proficiency is in most cases a marker of resistance, even though PARPi’s clinical activity in HR-proficient tumors has also been reported [51]. Most of the mechanisms of PARPi resistance come from studies on the few in vitro models selected for acquired resistance and on tumor samples from PARPi pretreated patients. However, to the best of our knowledge, no data are available on syngeneic cell lines; we therefore generated olaparib-resistant from murine HR -proficient and -deficient cells with stepwise increasing olaparib concentrations. The ID8 parental cell lines used for this study, obtained by CRISPR/Cas9 technology [24], are *TP53* (F3) deleted and, respectively, deleted/mutated in *TP53* and *Brca1* (*Brca1-/-*) and were selected as good in vitro models of OC.

The olaparib-resistant cell lines, both HR proficient and deficient, showed a 7- and 23-fold resistance to olaparib compared to the respective parental cells. This resistance lasted more than six months with no further drug treatment. No difference in cell growth was seen between parental and resistant cells. Olaparib caused a G2 block of the cell cycle in all the cells tested, which was less tight in resistant cells. This can be correlated with less DNA damage in both resistant versus parental cell pairs. In the case of ID8 OlaR, which expressed a much higher level of MDR1, it can be explained by the rapid drug efflux; in the case of *Brca1-/-* OlaR cells, it can be partially explained by reconstituted ability to repair DNA by HR.

Our data confirm the reported involvement of MDR1 pump efflux in olaparib resistance [52]. Its inhibition by verapamil caused a complete reversal olaparib resistance in F3 OlaR cells. The upregulation of MDR1 would explain the paclitaxel and doxorubicin cross-resistance. In addition to the fact that niraparib and rucaparib are MDR1 substrates too, no cross-resistance was found with these two PARPi. While the lack of cross-resistance with niraparib in F3 OlaR cells can be explained by its greater cellular permeability, the three-fold increase in sensitivity to rucaparib is still being studied. These data suggest that the re-challenge with PARPi can still be efficacious, and this is important considering that PARPis are approved in both first-line and recurrent settings and the use of PARPi after prior PARPi exposure will be much more common in the near future [53]. These data underlie the possibility to F3 OlaR cells do not show cross-resistance to DNA damaging agents (i.e., cisplatin and carboplatin), suggesting that pre-treatment with olaparib does not alter the subsequent response to platinum agents. This could be important as platinum drugs are used as second-line therapy in platinum-sensitive ovarian cancer, where olaparib can now be used in front-line maintenance setting. Similar data were reported in A2780 cells (HR proficient) made resistant to olaparib [35].

In the *Brca1-/-* OlaR HR deficient model, several different resistance mechanisms co-exist—partial restoration of HR, increased expression of MDR1 and a low PARP1 protein level. The partial restoration of HR is demonstrated by the increased HR/NHEJ repair ability ratio and by the increase in RAD51 foci formation after DNA damage. As previously reported [42], *BRCA1* reversion mutations have been associated with the functional reactivation of HR and have been reported both in cell lines and in PARPi pre-treated human tumor samples [54]. In the model engaged here, it is unlikely to be the case in *Brca1-/-* OlaR as exon 2 of *BRCA1* was deleted and this precludes a reversion mutation restoring the open reading frame of BRCA1 protein [24].

Partial restoration of HR might be partially explained by downregulation of *REV7*, which-with other NHEJ factors (i.e., 53BP1 and proteins of the Shieldin complex)-can enhance HR as well as SSA, i.e., homologous repair, by promoting end resection at the DNA double strand breaks, and has been associated with PARPi resistance [55,56,57]. The partial restoration of HR correlated with rucaparib and niraparib cross-resistance, supporting a common mechanism of action, but did not translate to platinum-based drugs cross-resistance. This lack of cross resistance has already been reported [58] and may possibly rely on different DNA repair mechanisms involved in the repair of DNA lesions induced by the drugs. While olaparib is in synthetic lethality with HR deficiency and inhibits DNA single strand repair, platinum DNA damage is repaired mainly by nucleotide excision repair (NER), HR, mismatch repair, NHEJ and translesion synthesis repair, all of which have been demonstrated to be involved in platinum resistance [59]. The sensitivity to UV damage was similar in *Brca1-/-* and *Brca1-/-* OlaR cells (data not shown) suggesting that NER, one of the most important repair pathways for UV damage and platinum drugs, was not affected during the acquisition of olaparib resistance and this might explain the similar platinum sensitivity in *Brca1-/-* and *Brca1-/-* OlaR, in spite of the partial restoration of HR repair.

Mutations in *PARP1* correlate with protein instability and PARPi resistance has been observed in different human tumors, including ovarian cancer [38]. *Brca1-/-* OlaR cells have a wild type *PARP1*; however, the protein level is lower than in *Brca1*-/- parental cells and the proteasome inhibition leads to PARP1 stabilization, suggesting faster protein degradation. The inhibition of the increased MDR1 mRNA expression was related to only a partial reversion of olaparib resistance.

These data strongly suggest that multiple mechanisms concur in olaparib resistance in the HR-deficient background. These data corroborate the results of the EVOLVE phase II study, which investigated the role of cediranib and olaparib combined after olaparib treatment progression, and found multiple mechanisms of olaparib resistance by whole-exome and RNA sequencing in the largest published post-PARPi patient population [60]. The heterogeneous and simultaneous resistant mechanisms described in ID8 *Brca1-/-* OlaR also align with recent data on PARPi-resistant *BRCA1-/-* retinal pigment epithelial cells, hTERT RPE-1 [61]. Those authors not only demonstrated that cyclic niraparib in vitro treatment of *BRCA1-/-* cells gives rise to multiple genetically and functionally heterogeneous PARPi-resistant clones, but that similar clonal heterogeneity was observed in a HGSOC patient’s tumor biopsy resistant to PARPi.

DDR inhibitors were more cytotoxic in *Brca1-/-* parental cells than in F3, confirming the reported greater dependency of HR-deficient cells on the ATR/Chk1/Wee1 axis [62,63]. There was cross-resistance in the OlaR cells, except for KU55933 (the ATM inhibitor). However, the combined treatments with olaparib are similarly synergistic in olaparib sensitive and resistant cell lines, in both a HR-proficient and -deficient background, suggesting that the effects of these combinations do not affect the mechanisms of resistance, and could be active in olaparib-resistant cases. The combination of ATRi and olaparib has already been shown to reverse PARPi resistance in in vitro and in vivo models with different mechanisms of resistance to PARPi [64]. While quite active in vitro, these combinations may, however, suffer from the fact that in a clinical setting they could give rise to cumulative haematological toxicities, as few reports have already noted [65,66]; this calls for schedule adjustments particularly in [platinum and olaparib] pre-treated patients, but nevertheless clinical investigation is warranted in olaparib-resistant cases.

Our study suffers some limitations. Even though ID8 cells are recognized as a validate mouse model of OC, they derive from mouse ovarian epithelia and may not represent the whole spectrum of human HGSOC which has been reported to originate also from fallopian cells. In addition, we focused on CRISPR/Cas9 technology and the ID8 *Brca1-/-*cell line we selected for olaparib resistance has a deleterious impairment of *Brca1* that could not be easily reversed, so we were somehow pushing versus permitting the full-spectrum of olaparib resistance mechanisms.

## 5. Conclusions

In conclusion, these data suggest that olaparib induced resistance in HR proficient and HR deficient cells rely on different mechanisms and seem to be more heterogeneous in a HR deficient background, possibly due to the greater instability of these cells. Nevertheless, combination of different DDR inhibitors is synergic in sensitive and resistant cells, regardless of the HR cell status. We think that our resistant models represent useful tools for the development of new therapeutic strategies to overcome olaparib resistance, including combination with immunocheckpoint inhibitors.

## Figures and Tables

**Table 1:** IC_50_ of different compounds in sensitive and olaparib resistant HR-proficient and -deficient ID8 cells. For statistical analyses unpaired *t*-test was used.

DRUG	F3	F3 OlaR	*p*	Fold Increase	*Brca1-/-*	*Brca1-/-* OlaR	*p*	Fold Increase
**Olaparib (µM)**	12.99 ± 2.31	88.59 ± 22.11	*p* = 0.0016	**6.9**	0.17 ± 0.028	3.94 ± 0.55	*p* < 0.0001	**23.1**
**Niraparib (µM)**	1.11 ± 0.22	1.00 ± 0.19	*p* = 0.94	**0.9**	0.065 ± 0.012	0.28 ± 0.035	***p* = 0.023 **	**4.3**
**Rucaparib (µM)**	12.5 ± 2.95	4.05 ± 0.61	***p* = 0.023 **	**0.3**	0.064 ± 0.015	1.03 ± 0.11	***p* = 0.0123 **	**15.6**
**Cisplatin (µM)**	19.32 ± 3.12	17.34 ± 6.61	*p* = 0.22	**0.9**	3.57 ± 0.91	4.26 ± 0.94	*p* = 0.30	**1.2**
**Carboplatin (µM)**	59.28 ± 5.46	59.19 ± 6.33	*p* = 0.94	**1**	25.51 ± 2.62	39.69 ± 4.90	*p* = 0.31	**1.4**
**ET-743 (nM)**	1.74 ± 0.37	2.04 ± 0.34	*p* = 0.85	**1.2**	0.24 ± 0.08	1.11 ± 0.29	***p* = 0.024 **	**4.6**
**Paclitaxel (nM)**	18.8 ± 2.21	55.66 ± 4.68	***p* = 0.039 **	**3**	28.1 ± 4.01	26.51 ± 3.37	*p* = 0.74	**0.9**
**Doxorubicin (nM)**	84.31 ± 20.42	657.3 ± 68.06	***p* = 0.035 **	**7.8**	34.63 ± 6.03	139.1 ± 23.25	***p* = 0.034 **	**4**
**AZD6738 (µM)**	0.30 ± 0.076	0.50 ± 0.06	***p* = 0.023 **	**1.7**	0.18 ± 0.03	0.4 ± 0.07	*p* = 0.059	**2.2**
**AZD7762 (µM)**	0.36 ± 0.064	1.56 ± 0.18	***p* = 0.0002 **	**4.3**	0.22 ± 0.04	0.46 ± 0.082	***p* = 0.037 **	**2.1**
**AZD1775 (µM)**	0.92 ± 0.14	0.99 ± 0.1	*p* = 0.66	**1.1**	0.35 ± 0.05	0.75 ± 0.10	***p* = 0.006 **	**3**
**KU55933 (µM)**	38.93 ± 15.17	26.56 ± 9.1	*p* = 0.51	**0.7**	21.43 ± 7.16	22.97 ± 6.03	*p* = 0.9	**1.1**

Bold represents the stress to the type of drug used, the fold increse of resistance and the signnificant *p* values.

## Data Availability

The data presented in this study are available on request from the corresponding author.

## Supplementary Materials

The following supporting information can be downloaded at: https://www.mdpi.com/article/10.3390/cancers14071807/s1, Figure S1: Graphical representation of olaparib resistance induction, Figure S2: Clonogenic assay of F3, F3 OlaR, *Brca1-/-* and *Brca1-/-* OlaR cells, Figure S3: Pharmacological characterization of F3, F3 OlaR, *Brca1-/-* and *Brca1-/-* OlaR cells, Figure S4: PARP1 expression in F3, F3 OlaR, *Brca1-/-* and *Brca1-/-* OlaR cells after olaparib treatment, Figure S5: DNA repair in olaparib-sensitive and -resistant *Brca1-/-*cells, Figure S6: Combination of ATR inhibitor and olaparib in ID8 cell lines, Figure S7: Full western blot figures and densitometry intensity ratio; Table S1: Primers used for RT-PCR and PARP1 sequencing, Table S2: Olaparib induced apoptosis. % of apoptosis detected by Annexin V and PI staining in olaparib sensitive and resistant cells at different time points after drug treatment.
