# Supplementary material for: Combinations of ATR, Chk1 and Wee1 Inhibitors with Olaparib Are Active in Olaparib Resistant Brca1 Proficient and Deficient Murine Ovarian Cells
Source: Cancers (Basel). 2022 Apr 1;14(7):1807. doi: 10.3390/cancers14071807 (PMC8997432; doi:10.3390/cancers14071807)
Supplement: Supplementary file 1 [file cancers-14-01807-s001.zip › cancers-1515540-supplementary.pdf]

cancers

Supplementary Materials

# Combinations of ATR, Chk1 and Wee1 Inhibitors with Olaparib are Active in Olaparib Resistant *Brca1* Proficient and Deficient Murine Ovarian Cells

**Michela Chiappa [1], Federica Guffanti [1], Martina Anselmi [1], Monica Lupi [1], Nicolò Panini [1], Lisa Wiesmüller [2] and Giovanna Damia [1,*]**

1 Department of Oncology, Istituto di Ricerche Farmacologiche Mario Negri IRCCS, 20156 Milan, Italy; michela.chiappa@marionegri.it (M.C.); federica.guffanti@marionegri.it (F.G.); martina.anselmi@unimi.it (M.A.); monica.lupi@humanitasresearch.it (M.L.); nicolo.panini@marionegri.it (N.P.)
2 Department of Obstetrics and Gynecology, Ulm University, 89075 Ulm, Germany; lisa.wiesmueller@uni-ulm.de
* Correspondence: giovanna.damia@marionegri.it

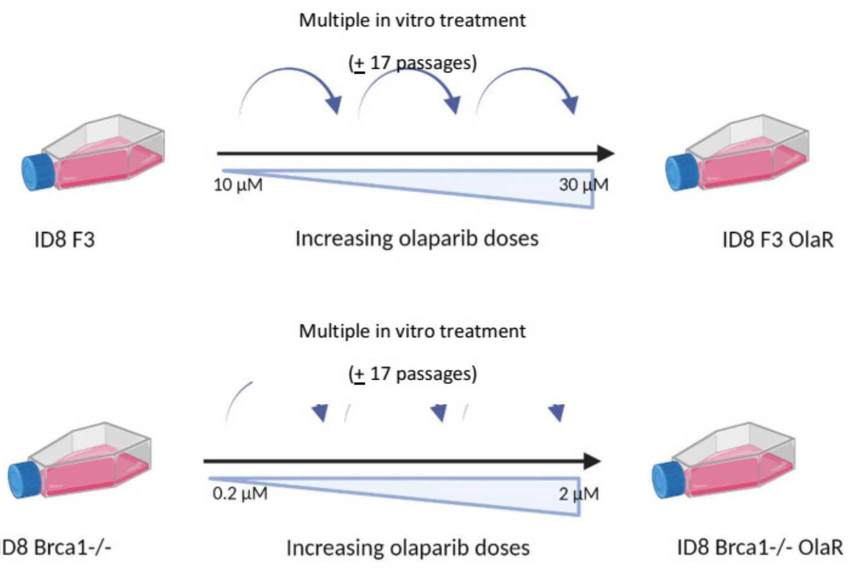


**Figure S1.** Graphical representation of olaparib resistance induction. ID8 F3 and *Brca1-/-* cells were treated with step-wise increasing doses of olaparib for six months to generate the corresponding resistant sublines. The image was created with Biorender.

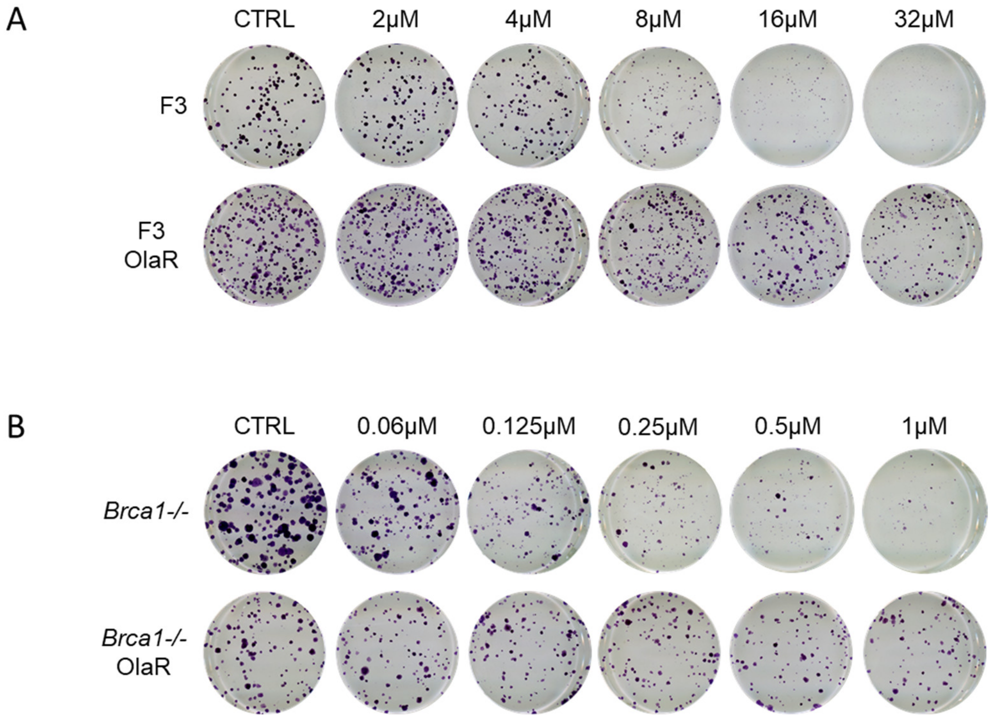


**Figure S2.** Clonogenic assay of F3, F3 OlaR, *Brca1-/-* and *Brca1-/-* OlaR cells. (**A**) Colony assay of ID8 F3 and F3 OlaR untreated and after olaparib treatment at 2 μM, 4 μM, 8 μM, 16 μM and 32 μM. (**B**) Colony assay of ID8 *Brca1-/-* and *Brca1-/-* OlaR untreated and after olaparib treatment at 0.06 μM, 0.125 μM, 0.25 μM, 0.5 μM and 1 μM.

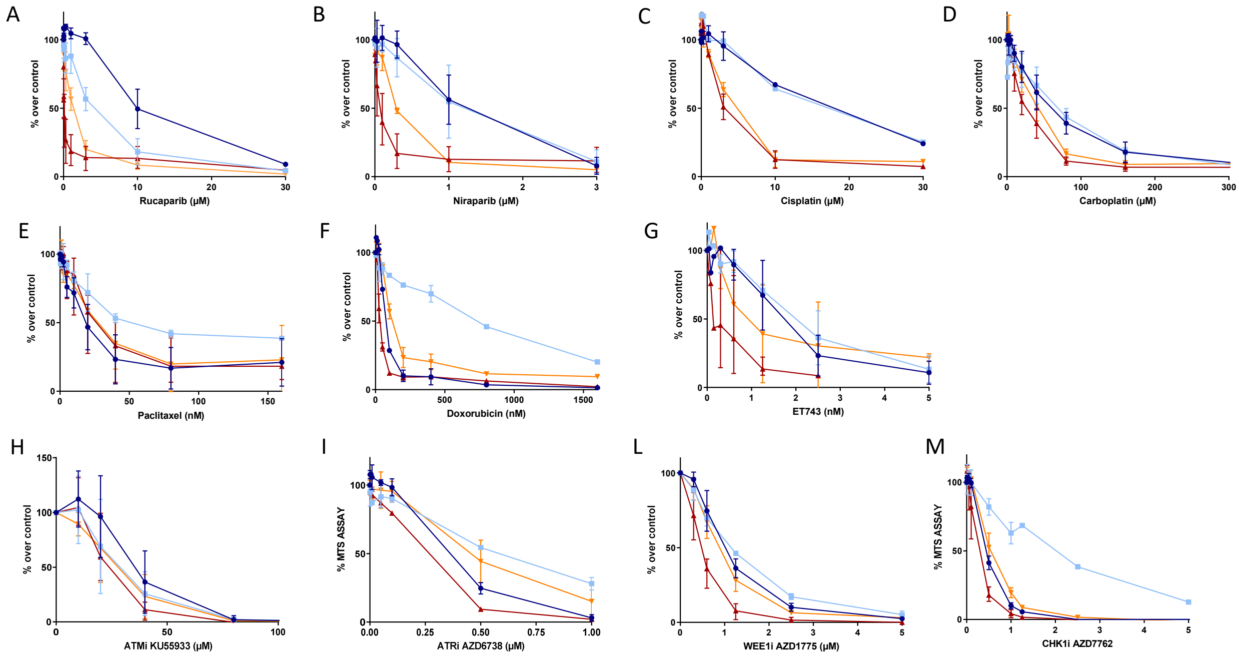


**Figure 3.** Pharmacological characterization of F3, F3 OlaR, *Brca1-/-* and *Brca1-/-* OlaR cells. (**A,B**) Dose response curve of PARPi rucaparib and niraparib in F3 (blue line), F3 OlaR (light blue line), *Brca1-/-* (red line) and *Brca1 -/-* OlaR cells (orange line). Data are the mean ± standard deviation (SD) of four independent experiments. (**C–G**) Dose response curve of anticancer agents cisplatin, carboplatin, paclitaxel, doxorubicin and ET-743 in F3 (blue line), F3 OlaR (light blue line), *Brca1-/-* (red line) and *Brca1 -/-* OlaR cells (orange line). Data are the mean ± SD of three independent experiments. (**H–M**) Dose response curve of ATMi, ATRi, Wee1i and Chk1i inhibitors in F3 (blue line), F3 OlaR

(light blue line), *Brca1*-/- (red line) and *Brca1* -/- OlaR cells (orange line). Data are the mean ± SD of three independent experiments.

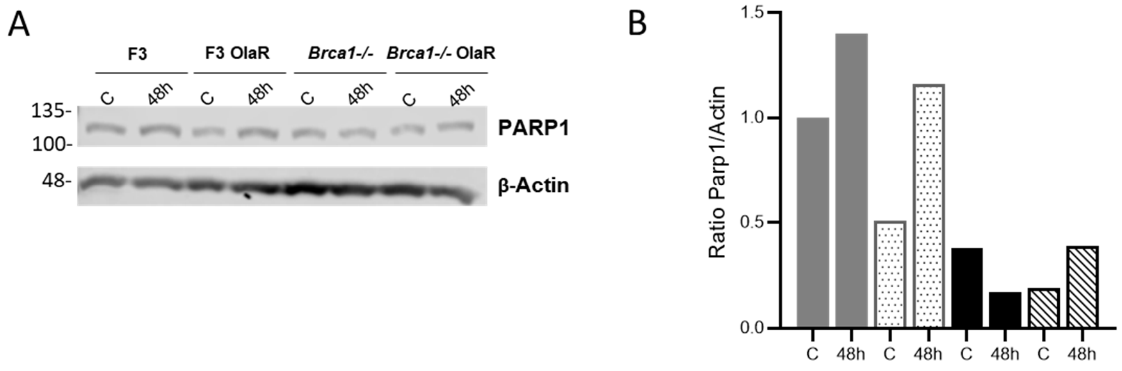


**Figure S4.** PARP1 expression in F3, F3 OlaR, *Brca1*-/- and *Brca1*-/- OlaR cells after olaparib treatment. (**A**) Western blot analysis of PARP1 expression after olaparib treatment at IC50 dose for 48h in F3 (filled grey column), F3 OlaR (patterned grey column), *Brca1*-/- (filled black column) and *Brca1*-/- OlaR cells (patterned black column). (**B**) Densitometric analysis of the western blot results expressed as the ratio of PARP1 to actin level.

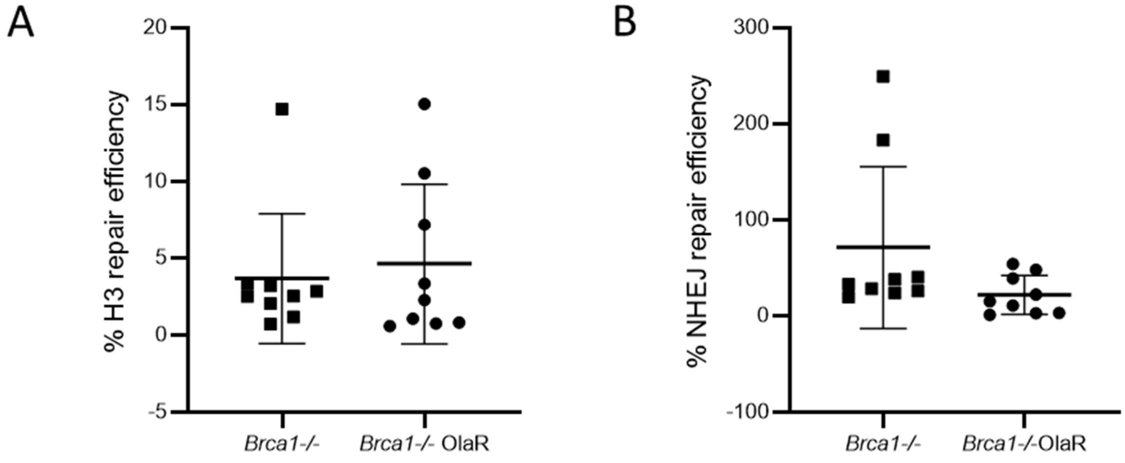


**Figure S5.** DNA repair in olaparib-sensitive and -resistant Brca1-/-cells. Basal levels of H3 (HR and SSA) (**A**) and NHEJ (**B**) repair by analysing pathway-specific DSB repair of GFP-based reporter plasmids EJ5SceGFP and HR-EGFP/3′EGFP transfected in the sensitive and resistant cells along with the I-*Sce*I meganuclease plasmid as detailed in Material and Methods. Each dot represent a single experimental point. For statistical analyses unpaired t test was used.

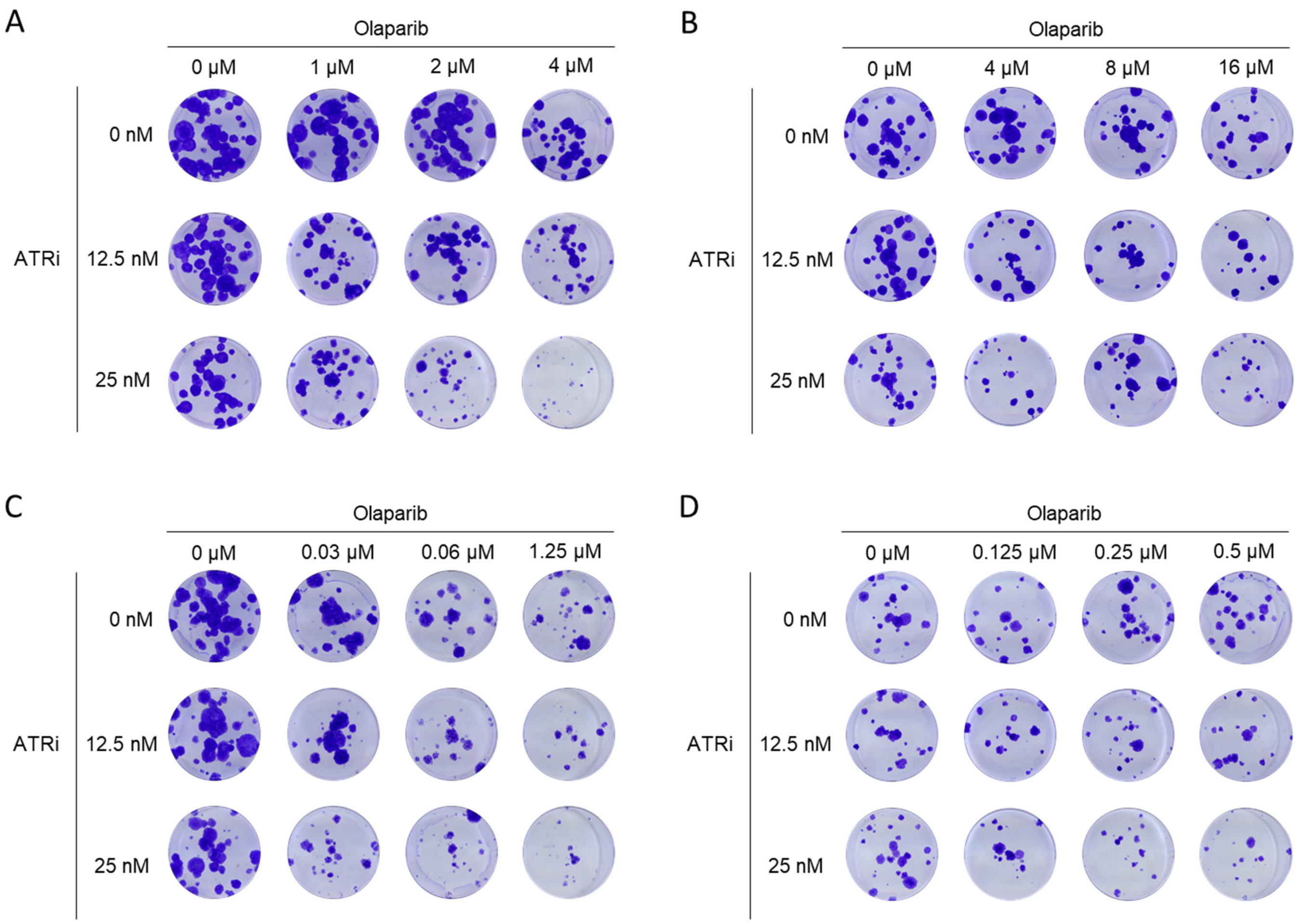


**Figure S6.** Combination of ATR inhibitor and olaparib in ID8 cell lines. Colony assay of ID8 F3 (**A**), F3 OlaR (**B**), *Brca1-/-* (**C**) and *Brca1-/-* OlaR (**D**) untreated and after combined treatment with olaparib and ATR inhibitor. .

A

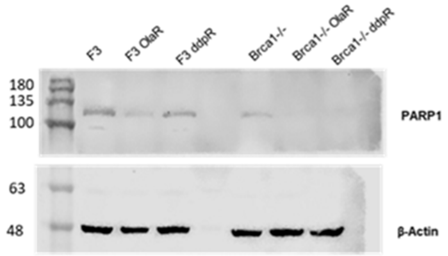


B

| | PARP1 AREA | ACTIN AREA | ratio/actin | vs parental |
|---|---|---|---|---|
| F3 | 16745154 | 14575619 | 1.15 | 1.00 |
| F3 OlaR | 5892083 | 10336497 | 0.57 | 0.50 |
| Brca1-/- | 9452447 | 12922033 | 0.73 | 1.00 |
| Brca1-/- OlaR | 859556 | 16625811 | 0.05 | 0.07 |

C

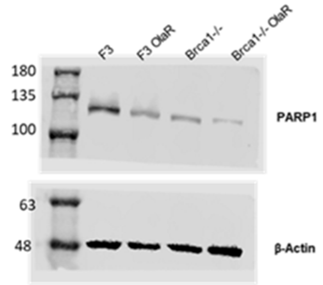


D

| | PARP1 AREA | ACTIN AREA | ratio/actin | vs parental |
|---|---|---|---|---|
| F3 | 30395045 | 24227167 | 1.255 | 1.00 |
| F3 OlaR | 11438196 | 17880924 | 0.640 | 0.51 |
| Brca1-/- | 7853125 | 21.483459 | 0.366 | 1.00 |
| Brca1-/- OlaR | 2148276 | 30316874 | 0.071 | 0.19 |

E

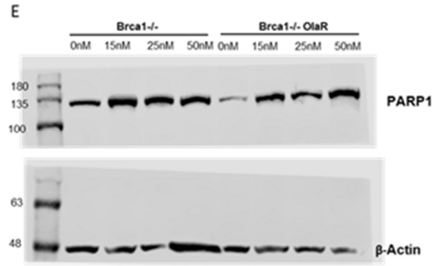


F

| | | PARP1 AREA | ACTIN AREA | ratio/actin | vs parental |
|---|---|---|---|---|---|
| Brca1-/- | 0 nM | 9322255 | 12561083 | 0.74 | 1.00 |
| | 15 nM | 16317255 | 9328083 | 1.75 | 2.36 |
| | 25 nM | 13964548 | 6018305 | 2.32 | 3.13 |
| | 50 nM | 13515205 | 27303489 | 0.50 | 0.67 |
| Brca1-/- OlaR | 0 nM | 1990598 | 9612477 | 0.21 | 0.28 |
| | 15 nM | 12643134 | 8904033 | 1.42 | 1.91 |
| | 25 nM | 9701619 | 7906548 | 1.23 | 1.65 |
| | 50 nM | 16715083 | 4263891 | 3.92 | 5.28 |

G

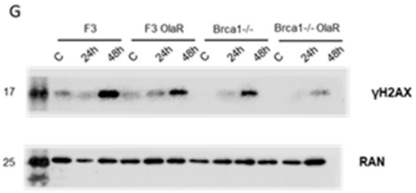


H

| | | γH2AX AREA | ACTIN AREA | ratio/RAN | vs ctrl |
|---|---|---|---|---|---|
| F3 | ctrl | 4236355 | 10059355 | 0.42 | 1.00 |
| | 24h | 2225406 | 3177284 | 0.70 | 1.66 |
| | 48h | 14697548 | 7280234 | 2.02 | 4.79 |
| F3 OlaR | ctrl | 3541062 | 6884113 | 0.51 | 1.00 |
| | 24h | 3940355 | 9571941 | 0.41 | 0.80 |
| | 48h | 8779891 | 7744820 | 1.13 | 2.20 |
| Brca1-/- | ctrl | 100000 | 4319820 | 0.02 | 1.00 |
| | 24h | 1554941 | 6682234 | 0.23 | 10.05 |
| | 48h | 7585477 | 8334477 | 0.91 | 39.32 |
| Brca1-/- OlaR | ctrl | 100000 | 6372355 | 0.02 | 1.00 |
| | 24h | 392335 | 5509820 | 0.07 | 4.54 |
| | 48h | 2740012 | 11253062 | 0.24 | 15.52 |

I

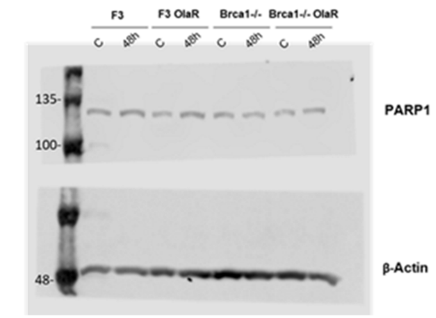


J

| | | PARP1 AREA | ACTIN AREA | ratio/actin | vs control | vs parental |
|---|---|---|---|---|---|---|
| F3 | ctrl | 10258962 | 10300276 | 1.00 | 1.00 | 1.00 |
| | 48h | 11434255 | 8163790 | 1.40 | 0.95 | 1.41 |
| F3 OlaR | ctrl | 3937477 | 7790154 | 0.51 | 1.00 | 0.51 |
| | 48h | 10743669 | 9301083 | 1.16 | 2.29 | 1.16 |
| Brca1-/- | ctrl | 6212184 | 16153518 | 0.38 | 1.00 | 1.00 |
| | 48h | 3524719 | 20893326 | 0.17 | 0.44 | 0.44 |
| Brca1-/- OlaR | ctrl | 3114598 | 16625619 | 0.19 | 1.00 | 0.49 |
| | 48h | 6058012 | 15645548 | 0.39 | 2.07 | 1.01 |

**Figure S7.** Full western blot figures and densitometry intensity ratio. (**A,C**) PARP1 protein expression in F3, F3 OlaR, *Brca1*-/- and *Brca1*-/- OlaR cells detected by western blot analysis and the corresponding densitometric analysis expressed as the ratio of PARP1 to actin level over the parental cell line (**B,D**). (**E**) PARP1 protein expression in *Brca1*-/- and *Brca1*-/- OlaR cells after treatment with different doses of the proteasome inhibitor PS341 and the densitometric analysis expressed as the ratio of PARP1 to actin level over *Brca1*-/-cell line (**F**). (**G**) γH2AX expression after olaparib treatment ($IC_{50}$ dose) at 24 and 48 hours and its densitometric quantification expressed as the ratio of γH2AX to RAN level over the control level (**H**). (**I**) PARP1 protein expression in F3, F3 OlaR, *Brca1*-/- and *Brca1*-/- OlaR cells after 48h olaparib treatment at IC50 doses detected by western blot analysis and the corresponding densitometric analysis expressed as the ratio of PARP1 to actin level over the control and over the parental cell line (**J**).

**Table S1.** Primers used for RT-PCR and *PARP1* sequencing. Sequences of the forward and reverse primers used to evaluate the gene expression by real-time-PCR and of the 7 couples of primers used for the sequencing of *PARP1* cDNA.

| GENE | PRIMER FORWARD | PRIMER REVERSE |
|---|---|---|
| *β-Actin* | GGCTGTATTCCCCTCCATCG | CCAGTTGGTAACAATGCCATGT |
| *PARP1* | CACTGCCCCTCTCTGTCAC | GCTTCGTCCTTGTTCTGGGA |
| *MDR1* | TGTCAGCTGGTATTTGGGCA | CAGTTCTGATGGCTGCTAAGAC |
| *REV7* | GGTGGTGGTGGTGATTTTGG | TCCACATGAGACAGGAGGGA |
| *TP53BP1* | TAGCCCGCTATCTGATGTGG | ACAGGGATGTCTTTGCTGGG |
| *SHLD1* | CAGGGAAGCAGCAACTCTCT | GTTCTCAGAGTCCCAGGAGC |
| *PARP* seq1 | GGAAACCGACACGTTAGC | TCGTCCCGCTTCTTGACAAA |
| *PARP* seq2 | ATGCGCCTGTCCAAGAAGAT | GGCAGTGACATCCCCAGTAC |
| *PARP* seq3 | GCAAGGAGTGTTCAGGCCA | CTCTGCCTTCACCTCAGCC |
| *PARP* seq4 | ATGGAGGAAGTGAAAGCGGC | GGCATTCCCAGTCTTCTCTTC |
| *PARP* seq5 | GATCTTCCGGTCCTGGGG | TGCGTTGTTCAGGAGTGGG |
| *PARP* seq6 | GGCAGCAGTGAATCCCAGAT | CTCCCTGAGATGTGTGGCAG |
| *PARP* seq7 | AGGGATCTACTTTGCCGACA | TTTTGTGGTGTCTGGTGCAG |

**Table S2.** Olaparib induced apoptosis. % of apoptosis detected by Annexin V and PI staining in olaparib sensitive and resistant cells at different time points after drug treatment.

| **Time (Hours)** | **F3** | | **F3 OlaR** | | |
|---|---|---|---|---|---|
| | **Ctrl** | **Ola 5 μM** | **Ctrl** | **Ola 5 μM** | **Ola 20 μM** |
| 0 | 4.96 | | 8.42 | | |
| 24 | 9.17 | 10.84 | 10.4 | 9.26 | 24.51 |
| 48 | 6.04 | 7.43 | 10.38 | 12.73 | 19.5 |
| **Time (hours)** | ***Brca1*-/-** | | ***Brca1*-/- OlaR** | | |
| | **Ctrl** | **Ola 0.75 μM** | **Ctrl** | **Ola 0.75 μM** | **Ola 7.5 μM** |
| 0 | 6.82 | | 6.06 | | |
| 24 | 5.06 | 7.35 | 9.63 | 21.06 | 14.15 |
| 48 | 7.31 | 15.16 | 7.1 | 15.12 | 16.31 |
